# Does public spending on sports improve people’s health? Empirical evidence from China

**DOI:** 10.3389/fpubh.2026.1744141

**Published:** 2026-03-03

**Authors:** Cheng Yang

**Affiliations:** Graduate School, Beijing Normal-Hong Kong Baptist University, Zhuhai, China

**Keywords:** mass sports, National fitness, physical exercise, public sports expenditure, residents’ health

## Abstract

**Background:**

Universal health is the foundation of human survival and a fundamental element for social and economic development. This study analyzes the theoretical basis of the impact of public sports expenditure on residents’ health, and based on the China Family Panel Studies data, uses a combination of macro and micro cross-sectional data to verify the relationship between public sports expenditure and residents’ health. It further explores the heterogeneous health effects of public sports expenditure from different groups’ perspectives.

**Methods:**

The micro data of the families involved in the study selected the 2014/2016 data of the China Family Panel Studies (CFPS) released by the China Social Science Survey Center of Peking University as the research sample. The Ordered Probit Model was used to analyze the impact effect of public sports expenditure on residents’ health.

**Results:**

Firstly, public sports expenditure and mass sports expenditure can effectively improve residents’ health, but other types of expenditures did not show significant results. This result remained valid after a series of robustness tests. Secondly, the health effect of public sports expenditure is heterogeneous. It significantly improves the health of high-income groups, low-education level groups, older adult groups, and rural groups. Finally, based on the empirical results, this study proposes corresponding policy recommendations to provide policy implications for managers to improve the health levels of different groups.

**Conclusion:**

This study finds that public sports expenditure has a positive impact on residents’ health, but the promotion effect of public sports expenditure on residents’ health levels still varies to some extent. Therefore, in the future, it is necessary to continue to improve sports infrastructure construction, expand the coverage of public sports venues and fitness facilities, and promote the open sharing of community and school sports facilities. At the same time, it is necessary to strengthen the supply of sports services for different groups, and ultimately achieve the universalization of health promotion effects.

## 1 Introduction

The health of all citizens is an important manifestation of a country’s comprehensive strength and a key indicator of social civilization and progress. In recent years, with the in-depth implementation of the “Healthy China 2030” planning outline, the national strategy of promoting fitness for all has been elevated to an unprecedented height (1). To smoothly implement the Healthy China strategy, the Communist Party of China and the state attach great importance to sports work, promote the development of sports, elevate national fitness to a national strategy, and promote the in-depth integration of national fitness and national health (2). In China’s 14th Five-Year Plan, it is explicitly proposed to “build a sports power,” promote the forward movement of the health focus, improve the public service system for mass fitness, advance the construction of social sports venues and facilities, gradually establish and improve the public sports service system, provide guarantees for mass fitness, and lay a solid foundation for a healthy China (3). Based on China’s strategic deployment on sports and national health, the government has been continuously increasing public sports spending. According to the data from the Central Budget and Final Accounts Disclosure Platform of the Ministry of Finance of China, the expenditure on public services for national fitness has increased from 13.281 billion yuan in 2006 to 50.764 billion yuan in 2020 over the past 15 years. The funds were mainly used in areas such as the construction of sports venues, mass sports activities, sports competitions, sports training, and sports diplomacy (4). The “2020 National Sports Venue Statistical Survey Data” shows that the number of sports venues in China has reached 3.7134 million, including 871,200 national fitness paths and 89,400 fitness trails (with a total length of 209,300 kilometers), and the total area of sports venues is 3.099 billion square meters. The per capita sports venue area has increased from 1.03 square meters in 2003 to 2.20 square meters, laying a solid venue foundation for the healthy development of residents’ leisure (5). From the perspective of the focus of public sports expenditure, the early development of public sports in China focused on competitive sports to enhance the country’s influence, the political and diplomatic functions of external image promotion, and to bring honor to the nation. During this period, public sports expenditure was mainly used for the construction of large competition venues, sports training and sports training venues (6). With the development of the economy and the continuous improvement of people’s lives. People’s demands for a better life have become more diversified and personalized. The pursuit of health has become even stronger. The sports industry has also adjusted its direction along with the development of society to meet the demands of people’s lives. Competitive sports need to coordinate the development of mass sports, attach importance to leading and catalyzing mass sports, and expand in diversified directions such as fitness and entertainment, education and aesthetics.

According to existing literature research, economic factors are important factors influencing residents’ health. Studies have shown that economic factors can enhance residents’ health levels and have a positive effect on their health (7). On the one hand, mainly with the development of the economy and the continuous improvement of public service facilities, residents have enjoyed a higher level of public services, thus enhancing their health. On the other hand, as the economic level rises, residents’ income also increases. The increase in residents’ income means that they can have better living conditions and nutrients. Therefore, the health level of the residents has been improved (8). In terms of policy analysis, scholars have, respectively, explored the extent to which policy implementation affects residents’ health from policy perspectives such as “urban and rural medical insurance” and “trade liberalization” (9). For example, Tang et al. (10) used the difference-in-differences model to study the impact of medical insurance policy pooling on the health of rural residents. The research results indicated that medical insurance policy pooling is conducive to improving the health level of rural residents. Chen and Wang (11) used the IV-probit model to study the impact of trade liberalization on residents’ health. However, the research results indicated that in regions where import tariffs were significantly reduced, the occurrence probabilities of hypertension, overweight and obesity among urban residents increased significantly, and the probability of good health decreased significantly. Relevant studies have explained from different perspectives that residents’ health is influenced by various macro policies and economic environments. However, sports, as a means of health investment, are both economical and effective (12). At present, there are relatively few studies on the relationship between public sports expenditure and residents’ health, especially lacking relevant theoretical foundations. There is a lack of theoretical basis for the direction of public sports expenditure that affects residents’ health. Therefore, this study explores why public sports expenditure affects residents’ health, attempting to discover its influencing mechanism and provide theoretical expansion for the impact of public expenditure on residents’ health. The research breaks down public sports expenditure to explore whether the effects of each part of the expenditure on residents’ health are different. Conduct a classified study on the health heterogeneity effects of different groups, attempting to discover the health heterogeneity effects of public sports expenditure on various groups. Provide theoretical samples for the research on the impact of public sports expenditure on residents’ health.

## 2 Literature review

### 2.1 Research on the impact of public health expenditure on residents’ health

As medical and health care is the most direct factor affecting residents’ health, public health expenditure is an important indicator for measuring the level of medical and health care. Therefore, the impact of public health expenditure on residents’ health has also become a hot topic of scholars’ research (13). However, based on the research results of the existing literature, there is no unified conclusion on the relationship between public health expenditure and residents’ health. Some scholars believe that public health expenditure has a promoting effect on residents’ health, and increasing public health expenditure can promote the improvement of residents’ health levels (14). Some research results also show that because the scale of government healthcare expenditure also has a spatial spillover effect, the state continuously invests in the public sector of healthcare. However, it is not the case that the more the government invests in the medical industry, the higher the health level will definitely be (15).

### 2.2 Research on the impact of public education expenditure on residents’ health

Most studies have confirmed that expanding public education expenditure can enhance the national education level (16). The higher the educational level of residents, the healthier their behaviors will be and the better their socio-economic conditions will be. Therefore, residents will have a higher level of health. Public education expenditure has a certain regulatory effect on the health benefits of education. Public education expenditure can indirectly enhance the health level of residents by improving their educational attainment. Moreover, the lower the educational level of the residents, the more obvious the indirect regulatory effect of public education expenditure will be. However, the impact of public education expenditure on the health of residents with different household registrations varies. For rural residents, higher public education expenditure does not necessarily promote the health level of residents. Only in economically developed rural areas can higher public education expenditure promote the health level of residents (17).

### 2.3 Research on the impact of social security expenditure on residents’ health

Social security expenditures mainly consist of expenditures on social insurance, social assistance, social welfare and other aspects. Social security is used to safeguard citizens’ basic living rights and interests. It reduces the income gap among different groups through secondary income distribution and ensures citizens’ expenditures in medical care, basic living and other aspects. Therefore, social security expenditure is to enhance residents’ living standards and their willingness to seek medical treatment through the redistribution of income, thereby improving their health levels. Meanwhile, the health effects of social security expenditures vary among different groups (18). Research has found that social security expenditures have a significant health effect on economically disadvantaged groups and the older adult. Compared with high-income groups, low-income groups are more dependent on social security expenditures and thus have a more significant correlation (19). Due to the current global aging situation, the older adult are the main audience for social security expenditures. The older adult population is a pure consumer population, and their health conditions are greatly influenced by social security expenditures.

### 2.4 Research on the impact of public environmental expenditure on residents’ health

Relevant research has found that public environmental expenditure is conducive to improving residents’ health (20). In terms of improving environmental quality, the government mainly makes financial inputs in two aspects: pollution control and environmental protection. Among them, environmental protection expenditures are more conducive to reducing pollution and improving environmental quality (21). The improvement of environmental quality brings about a cleaner ecological environment and a healthier lifestyle. A clean environment itself is beneficial to residents’ health. In a beautiful environment, people will engage in more outdoor activities, further enhancing their health.

### 2.5 Research on the impact of public sports expenditure on residents’ health

The existing research mainly explores the issue of public sports expenditure from several perspectives such as scale and efficiency. From 1998 to 2021, the absolute scale of public sports expenditure in China has been on the rise. However, in terms of its proportion to the gross domestic product, its relative scale is decreasing (22). At present, the overall scale of public sports expenditure in China is relatively small. When compared with public education expenditure, public science and technology expenditure, and public medical and health expenditure, the proportions are all too small. Especially when compared with developed countries, there is still a considerable gap (23). Meanwhile, existing literature also studies the impact of sports facilities on residents’ health from the perspective of sports facilities and finds that convenient community sports infrastructure is conducive to improving residents’ health. However, there is an imbalance between supply and demand in public sports infrastructure, which is not conducive to improving residents’ health (24).

From the existing research results, there are two different views on the impact of public expenditure on residents’ health. On the one hand, public expenditure can significantly enhance the health level of residents. This result is generally believed to be that increasing public expenditure can optimize infrastructure construction, thereby facilitate residents’ lifestyles and improve their health levels. Krekel et al. (25) estimated the impact of the London Olympics on residents’ health. Through the use of the difference-in-differences model, it was calculated that the hosting of the London Olympics reduced the intake of substances such as alcohol and improved the health levels of residents. This further verified the aforementioned conjecture. On the other hand, scholars believe that simply increasing public expenditure cannot continuously improve the health level of residents, which is related to the operational capacity of public administrative departments. The same result is also reflected in public sports expenditure. Steckenleiter et al. (26) conducted a study on the impact of public expenditure on sports in Germany on the level of sports participation. Through geographical distance analysis, it was found that there was no significant improvement in sports participation among residents of different age groups.

Based on this, this paper utilizes the sample data of residents from 25 provinces in China, and the research subjects cover people with different characteristics in the three age groups of the older adult, middle-aged people and young people. Regression is conducted on the overall expenditure and each part of the expenditure of public sports expenditure on the health of residents, and further analyze the impact of public sports expenditure on urban and rural areas, residents of different incomes and different age groups, attempting to explore the relationship and mechanism between public sports expenditure and the health level of residents.

## 3 Theoretical analysis and research hypotheses

### 3.1 The overall impact of public sports expenditure on residents’ health

The theory of responsible government holds that when the people grant it rights, the government must fulfill its duties and obligations well. Therefore, the government must actively respond to the demands of the people, take effective measures, and earnestly solve the problems that concern the people to ensure the interests of society (27). Guided by the theory of responsible government, improving the health level of residents is a major responsibility of the government. The government’s investment in public sports, provision of public goods to residents, and guarantee of residents’ right to enhance their own health through participation in physical exercise activities are manifestations of the government’s fulfillment of its duty to improve health (28). Firstly, the primary function of public sports expenditure is to provide tangible infrastructure and services. This involves building and maintaining physical spaces such as public sports facilities, community fitness trails, and park green spaces, as well as organizing mass sports events and community sports activities. These investments directly increase the supply of opportunities for residents to engage in physical exercise (29). At the same time, to ensure that these facilities operate effectively and encourage residents to continue using them, there needs to be implicit policies and institutional guarantees. This includes formulating scientific fitness plans, enacting laws and regulations to ensure the allocation of land for public sports facilities and their openness, establishing a system of social sports instructors, and conducting publicity on sports health knowledge. These intangible services provide rules, guidance and motivation for the effective utilization of the physical facilities, ensuring that public investments are not left idle or ineffective (30). Therefore, the combination of tangible sports facilities and intangible sports services jointly influence the health-related behaviors of residents. Convenient facilities reduce the actual difficulties in exercising, while scientific guidance and an active community sports atmosphere can enhance residents’ motivation and skills for exercise (31). Finally, changes in physical activity lead to healthy outcomes. Regular scientific physical exercise can directly enhance physical fitness, prevent chronic diseases, and relieve mental stress, thereby steadily converting participation in sports into the accumulation and improvement of personal health capital.

*Hypothesis 1*: Public sports expenditure can enhance the health level of residents.

### 3.2 The heterogeneous impact of public sports expenditure on residents’ health

Relevant studies have shown that the degree of impact of public sports expenditure on residents’ health is associated with group characteristics, urban and rural regional development, and income levels (32). There are differences in age, educational level and economic level among social groups, which may lead to different impacts of public sports expenditure on the health levels of different social groups. Some studies have found that public expenditure has a more obvious promoting effect on the health level of the middle-aged group, and has a greater impact on the sense of social fairness of the middle-aged and older adult, those with lower educational levels, and the upper-middle class. The improvement effect on groups with primary school education and below is relatively weak (33). From the perspective of the health production function, groups with a higher socioeconomic status tend to have a stronger ability to transform health. Not only can they make more effective use of public sports resources, but they also usually have better health knowledge and more active lifestyle choices, which enables the same level of public sports investment among these groups to generate greater health returns. On the contrary, although disadvantaged groups may have higher health needs, they are often limited by their ability to access resources and health literacy, and thus find it difficult to fully convert public sports services into actual health benefits (34). Meanwhile, there are practical disparities in economic development levels and sports facilities resources between urban and rural areas, which may lead to differences in the impact of public sports expenditure on residents’ health levels. Such differences are closely related to the long-standing urban–rural dual system in China (35). Cities have always been the focus of social public service resource allocation, while the proportion of public service resources in rural areas is relatively small, resulting in higher public expenditure in towns and cities, which may promote more obvious health effects. In addition, external differences such as geographical environment, resource environment and policy orientation in regional development may also lead to differences in the impact of public sports expenditure on residents’ health.

*Hypothesis 2*: Public sports expenditure has heterogeneous effects on residents’ health.

## 4 Research design

### 4.1 Selection and explanation of variables

#### 4.1.1 The explained variable

The explained variable in this article is the health of residents (*Health*). Self-rated health was adopted from the China Family Panel Studies (*CFPS*) database released by the Center for Chinese Social Sciences Survey of Peking University (36). The relevant question is on page 502 of the questionnaire: How do you think your health condition is? There are five levels of answers. It should be noted that self-assessed health can better reflect the health of residents at the micro level. It is generally believed that the early changes in the body cannot be well detected by medical means. And individual residents can judge whether their physical condition has changed through their own feelings. This self-perceived change can be reflected through self-assessment of health. Self-assessed health also represents the residents’ own understanding and perception of the severity of the disease, and even implies important contents closely related to the individual’s health change trajectory, such as the family medical history of the interviewed residents, making it more comprehensive and all-round. At the same time, self-assessed health has a relatively high correlation with both physical health and mental health. It can comprehensively reflect the health of residents. Therefore, this article selects self-assessed health as the standard for measuring the health of residents.

#### 4.1.2 Explanatory variable

The core explanatory variable of this study is public sports expenditure, which is the sports section of Culture and Sports Media under the classification of expenditure in the “Statistical Yearbook of China’s Sports Industry.” It is the total budget expenditure of the central and local governments for sports undertakings in each province. As the expenditure on sports in culture, sports and media accounts for nearly 80% of the total expenditure of the sports system, and mass sports and sports venues in the expenditure on culture, sports and media are the parts that most directly affect the health of the masses, competitive sports are the factors that affect the mental health of the public. Therefore, the expenditure on sports in culture, sports and media was selected as the explanatory variable for public sports expenditure in this study. In addition, based on relevant literature and the expenditure classification of the national sports system’s public budget in the “China Sports Statistical Yearbook,” public sports expenditures are classified according to their impact on residents’ health: Expenditure on competitive sports (sports competitions, sports training), sports venues, mass sports, and other expenditures (administrative operation, general administrative management affairs, agency services, sports project management, sports exchanges and cooperation, etc.). The specific indicators are: per capita expenditure on public sports (*Physical*), per capita expenditure on mass sports (*Amateur*), per capita expenditure on sports venues (*Gym*), per capita expenditure on competitive sports (*Athletic*), and per capita expenditure on other expenditures (*Else*).

#### 4.1.3 Control variable

Referring to existing research, both individual and social factors that affect residents’ health are taken as control variables (37, 38).

(1) Government healthcare expenditure (*Medical*). In China, there are differences in the medical expenditure capacity among local governments, and the scale of medical expenditure varies among provinces. The degree and effect of its impact on the health of residents in different regions vary. Therefore, choosing healthcare expenditure as the control variable can control the impact of public healthcare on the health level of residents.
(2) Medical insurance (*Insurance*). This article summarizes based on the answers to the medical insurance type questionnaire in CFPS. The new rural social endowment insurance, basic endowment insurance, supplementary endowment insurance for enterprises, commercial endowment insurance, retirement benefits received from the current government agency or public institution after retirement, rural endowment insurance (old rural endowment insurance), and urban resident endowment insurance will be assigned a value of 1, and no value will be assigned to 0.
(3) Per capita GDP (*GDP*). Per capita GDP reflects the living standards and economic development levels of each region at that time. It is generally believed that in economically developed regions of our country, public services and facilities are more complete and have a higher utilization rate, and people’s health levels are also higher. GDP is a crucial factor influencing the expenditure on various public services, which in turn affects the intensity of these services, thereby influencing the production and lifestyle as well as the health conditions of residents. It is a factor that has a significant impact on the health of residents.
(4) Urban and rural residents (*City*). The urban and rural classification of the location where the surveyed subjects were at that time in the data of the National Bureau of Statistics was adopted in the CFPS. It is generally believed that urban residents are healthier than rural residents. Cities usually gather a large number of public resources, such as medical care, education, transportation, etc. The dual structure of urban and rural areas in China has led to differences in the distribution of public resources. Coupled with the promotion of urbanization, the disparity between urban and rural areas has intensified. Under the guidance of the distribution system that prioritizes efficiency while taking fairness into account, rural areas are vast and sparsely populated, resulting in relatively fewer public service resources, which can affect residents’ health. Therefore, the urban–rural factor has been included as a control variable.
(5) Environmental factors (*Pollution*). The degree of environmental pollution is measured by the per capita emission of sulfur dioxide in each province. It is generally believed that air pollution is the main factor causing respiratory diseases in people and is not conducive to the health of residents.
(6) Drinking water source (*Water*). Whether water sources are clean or not is directly related to people’s health. This article classifies water sources by selecting cooking water from CFPS. Well water, river water, lake water, pond water/mountain spring water, cellar water, rainwater, and other water that has not undergone filtration treatment are classified as natural water and are assigned a value of 1. Tap water, bottled water/purified water/filtered water are used as processing water and are assigned a value of 0.
(7) Age (*Age*). It is generally believed that the health stock decreases with age. Age is one of the important factors affecting health.
(8) Gender (*Gender*). It is generally believed that men are healthier than women. The physiological and pathological differences between men and women are one factor contributing to the disparity in health between the two genders. Another reason is that women often underestimate their own health due to physiological reasons. Therefore, gender needs to be included as a control variable in the study.
(9) Marriage (*Marriage*). It is generally believed that whether a marriage is happy is an important factor affecting the health of family members.
(10) Income (*Income*). Adopt the per capita net household income in CFPS. Per capita net household income can most effectively reflect an individual’s available income and consumption capacity. It is generally believed that groups with higher incomes have more social interactions and pay more attention to their appearance and grooming. Through exercise, they can achieve better looks. And social skills can be enhanced through exercise. Therefore, income is included as a control variable.
(11) Educational attainment (*Edu*). Education is an investment in health. People with a higher level of education tend to invest more in education, and accordingly, their health output will also be higher.
(12) Whether one smokes or not (*Smoke*). This article uses the question from CFPS: Have you smoked recently? To be used as a control variable.
(13) Excessive drinking (*Drink*). The impact of alcohol on people’s health is also significant. Moderate drinking is beneficial to physical health, while excessive drinking can harm the health of residents. In this paper, whether one drinks alcohol more than three times a week in CFPS is selected as the control variable to measure whether drinking is excessive.

### 4.2 Measurement model

Liu and Huo (39) regarded health capital as a part of human capital, constructed a health production function model, and theoretically derived the main factors affecting health. This model assumes that individuals inherit a certain amount of health stock, which decreases with age but can be increased through investment. In this model, the determinants of health include past health conditions, access to medical services, and personal factors. It should be noted that in the main regression section, the health level of the explained variable uses the 2016 data from the CFPS, and the explanatory variable for public sports expenditure uses the 2016 “Statistical Yearbook of Sports Affairs.” In the robustness test section, through lag variables and instrumental variable tests, the public sports expenditure data for 2014 and 2015 is used, and the explained variable still uses the 2016 data from the CFPS (40). This paper constructs the following mixed cross-section Ordered response econometric model:


$${\text{Health}}_{\mathrm{it}}=\alpha +\beta_{1}\text{Physica}l_{\mathrm{it}}+\beta_{2}{\text{Control}}_{\mathrm{it}}+\varepsilon_{\mathrm{it}}$$ (1)

Among them, i and t represent personal codes and years, respectively. β_1_ and β_2_ are the coefficients to be estimated. Health_it_ is an indicator of residents’ health and the explained variable. Physical_it_ is an indicator of public sports expenditure and serves as the core explanatory variable. Control_it_ is the set of control variables, and ε_it_ is the random disturbance term.

### 4.3 Data sources and descriptive statistics

The microdata of households involved in this study mainly come from the 2014/2016 data of the China Family Panel Studies (CFPS) released by the Center for Social Sciences Survey of Peking University. The macroeconomic indicators and air pollution indicators mainly come from the “China Statistical Yearbook.” The data on public sports expenditure are from the “Statistical Yearbook of Sports” of 2014, 2015 and 2016. It should be noted that in the principal regression section, the health level of the explained variable is based on the 2016 data from CFPS, and the public sports expenditure of the explained variable is based on the 2016 “Statistical Yearbook of Sports.” In the robustness test section, through lag variables and instrumental variable tests, the public sports expenditure data for 2014 and 2015 is used, and the dependent variable still uses the 2016 data from the CFPS. Meanwhile, public sports expenditure refers to the sports section under “Culture, Sports and Media” in the national sports system’s public budget expenditure as recorded in the “Statistical Yearbook of Sports.” Ultimately, 53,031 initial observations were obtained. Table 1 shows the descriptive statistical results of each variable.

**Table 1:** Descriptive statistics of each variable.

Variable	Variable symbol	Mean	SD	Max	Min
Explained variable	Health	3.687	1.142	5	1
Core explanatory variable	Physical	0.366	0.168	1.255	0.010
	Amateur	0.167	0.100	0.324	0.014
	Gym	0.089	0.342	0.512	0.009
	Athletic	0.033	0.075	0.009	0.307
	Else	0.175	0.037	0.867	0.008
Control variable	Age	42.354	15.977	98	16
	Gender	0.802	0.385	1	0
	Marriage	0.765	0.248	1	0
	Income	9.611	1.086	16.375	−1.385
	City	0.354	0.511	1	0
	Smoke	0.432	0.312	1	0
	Drink	0.132	0.298	1	0
	Water	0.352	0.501	1	0
	Medical	6.895	0.137	6.032	7.852
	Insurance	0.698	0.384	1	0
	Pollution	20.787	9.364	60.721	5.341
	GDP	9.675	0.341	12.242	11.824
	Edu	2.637	1.220	8	1

## 5 Research results and analysis

### 5.1 The overall impact of public sports expenditure on residents’ health

This study first conducts a correlation test between public sports expenditure and classified expenditure by purpose on residents’ health to measure the impact of total expenditure and each classified expenditure on residents’ health. According to the measurement model in Equation 1, Table 2 presents the test results of the correlation between per capita public sports expenditure in China and residents’ health. It can be seen that only Models 1 and 2 passed the 1% test, showing a significant positive correlation, which validates Hypothesis 1 and indicates that public sports expenditure and mass sports expenditure can enhance residents’ health. This further validates the research findings of Wang et al. (23), which states that increasing public expenditure on sports can boost residents’ sports consumption and thereby improve their health. Since the expenditure on mass sports mainly consists of the expenses related to mass sports activities such as national fitness, it is the expenditure directly used for the health output of the masses and also the expenditure with the direct purpose of improving the health of residents. Expenditure on mass sports is mainly used for the development of mass sports activities and the construction of infrastructure, etc. This can effectively increase the frequency of residents’ physical exercise and promote their health. Therefore, the positive effect is obvious. The effective improvement of residents’ health by mass sports expenditure also reflects the achievements of the national fitness program in enhancing residents’ health. It is a positive outcome of the mutual coordination of tangible and intangible services provided by the state through infrastructure construction and national development strategies. Except for the expenditure on mass sports, the regression results of the other several types of expenditure did not show a significant level. The reasons for this might be as follows. First, the expenditure on sports venues mainly consists of the construction costs and maintenance expenses of sports venues in various regions. Large sports venues are mostly used for hosting major sports events and are usually idle or even abandoned. Large sports venues that are idle have failed to make full use of their convenience for the public. Moreover, the construction of sports venues also occupies the living space of residents, increasing the cost of living and exercise, which is not conducive to improving the health level of residents. Second, expenditures on competitive sports are used for comprehensive sports meetings and individual sports competitions, training subsidies for sports teams at all levels, and the purchase of equipment, etc. It has no direct impact on the health of residents. Thirdly, other expenditures mainly consist of the salaries of sports system staff, project management fees, and logistics expenses. The expenditure of the sports system has failed to effectively provide public sports services that can enhance the health of residents, which is probably strongly related to the stage of the sports system’s construction.

**Table 2:** Overall impact results of public sports expenditure on residents’ health.

Variable	(1)	(2)	(3)	(4)	(5)
Physical	0.153*** (6.55)				
Amateur		0.732*** (8.95)			
Gym			0.175 (0.17)		
Athletic				0.127 (0.73)	
Else					0.012 (0.75)
Age	−0.252*** (−9.54)	−0.139*** (−5.27)	−0.337*** (−9.97)	−0.846* (−1.76)	−0.702* (−1.58)
Gender	0.132*** (3.14)	0.113*** (3.99)	0.133*** (3.46)	0.133*** (3.46)	0.119*** (3.07)
Marriage	−0.065*** (−9.66)	−0.052*** (−7.09)	−0.055*** (−9.98)	−0.059*** (−5.98)	−0.061*** (−6.32)
Income	0.024*** (6.54)	0.022*** (6.57)	0.022*** (6.52)	0.023*** (6.66)	0.024*** (6.54)
City	−0.053*** (−5.21)	−0.050*** (−5.27)	−0.051*** (−5.20)	−0.050*** (−5.27)	−0.051*** (−5.20)
Smoke	0.033*** (4.15)	0.031*** (4.21)	0.033*** (4.15)	0.032*** (4.07)	0.032*** (4.07)
Drink	0.132*** (6.33)	0.134*** (6.32)	0.133*** (6.30)	0.132*** (6.27)	0.131*** (6.25)
Water	−0.056*** (−8.76)	−0.055*** (−8.77)	−0.054*** (−8.70)	−0.055*** (−8.77)	−0.054*** (−8.69)
Medical	−0.088*** (−5.56)	−0.087*** (−5.55)	−0.087*** (−5.57)	−0.088*** (−5.52)	−0.087*** (−5.55)
Insurance	−0.031*** (−6.26)	−0.029*** (−6.23)	−0.030*** (−6.18)	−0.031*** (−6.26)	−0.032*** (−6.17)
Pollution	0.004*** (7.23)	0.003*** (7.09)	0.003*** (7.14)	0.005*** (7.06)	0.003*** (7.09)
GDP	0.129*** (4.43)	0.098*** (4.88)	0.114*** (4.86)	0.132*** (4.77)	0.095*** (4.65)
Edu	0.023*** (5.39)	0.022*** (5.52)	0.023*** (5.30)	0.021*** (5.32)	0.022*** (5.33)
*N*	53,031	53,031	53,031	53,031	53,031
*R* ^2^	0.685	0.685	0.686	0.687	0.687

***, **, * represent significant at the 1, 5, and 10% levels, respectively.

### 5.2 The marginal effect of public sports expenditure on residents’ health

To enhance the interpretability of the benchmark regression results, it is necessary to calculate the marginal effects of each parameter in order to better measure their impact on residents’ health. Table 3 reports the marginal effect values of each variable when the dependent variable takes different values. Based on the marginal effects, the core explanatory variable, public sports expenditure, shows a negative impact only in the “unhealthy” sample. For every increase of one unit in *Physical*, the probability of residents being “unhealthy” decreased by 0.109, the probability of being “average” increased by 0.031, the probability of being “relatively healthy” rose by 0.065, the probability of being “very healthy” increased by 0.052, and the probability of being “extremely healthy” rose by 0.034. Among the other sub-dimensions of public sports expenditure, only the public sports expenditure for the masses showed a significant increase. This indicates that the primary effect of public investment in sports is not to merely slightly improve the health of all individuals by one level, but rather to significantly reduce the probability of unhealthy conditions and increase the probability of healthy conditions, thereby promoting a shift in the overall health distribution towards a higher level.

**Table 3:** The marginal effects of each variable.

Variable	(1) Unhealthy	(2) Average	(3) Relatively healthy	(4) Very healthy	(5) Extremely healthy
Physical	−0.109*** (−4.64)	0.031*** (3.75)	0.065*** (4.53)	0.052*** (4.06)	0.034*** (3.95)
Amateur	−0.193*** (−5.23)	0.043*** (6.32)	0.055*** (5.44)	0.046*** (4.52)	0.041*** (5.31)
Gym	−0.012 (−0.51)	0.035 (0.47)	0.182 (0.51)	0.045 (0.93)	0.124 (0.55)
Athletic	−0.002 (−0.42)	0.242 (0.44)	0.042 (0.93)	0.112 (0.84)	0.125 (0.83)
Else	−0.031 (−0.43)	0.064 (0.74)	0.184 (0.42)	0.524 (0.62)	0.169 (0.47)

***, **, * represent significant at the 1, 5, and 10% levels, respectively.

### 5.3 Heterogeneous effects of public sports expenditure on residents’ health

The overall regression results show that only by increasing expenditure on public sports can the physical health of residents be effectively improved. It should be noted that the reason why large-scale public sports expenditures can reveal significant heterogeneity effects is that they go beyond the stage of universal provision and touch upon the structural differences in resource utilization efficiency, acquisition capabilities, and behavioral substitution among different groups. The allocation of public resources is not completely homogeneous. Large-scale public sports expenditure projects, such as large sports parks and comprehensive gymnasiums, may have implicit structural biases in their location selection and functional design, favoring the service of specific groups or regions. While small-scale public sports expenditures have a limited impact range. Large-scale new investments in location selection and functional design will become a process of resource redistribution, which may amplify the differences in geographical accessibility and information acquisition capabilities among different groups. The results of the above-mentioned overall regression are only average and mask the possible heterogeneous effects on the health of residents with different characteristics. Analyzing the differences in the impact of mass sports expenditure on the health of different groups can more effectively enhance the health benefits of mass sports expenditure. For this reason, this study further divided the entire sample into various sub-samples based on different income groups, different educational groups, different age groups, and whether they live in urban or rural areas, to compare whether the positive effect of mass sports expenditure on residents’ health varies due to different samples.

#### 5.3.1 The impact of public sports expenditure on the health of different income groups

As shown in Table 4, public sports expenditure has indeed produced different health effects on residents of different income groups. For high-income groups, the regression coefficient of public sports expenditure is positive and has passed the 10% significance level test statistically. This indicates that public sports expenditure has a relatively significant positive promoting effect on such residents. This result is consistent with the conclusion of previous literature (41). High-income groups, due to the need for social interaction, pay more attention to their appearance and grooming. They can maintain a good appearance through exercise. And social skills can be enhanced through exercise. Therefore, they pay more attention to exercise and have a higher level of physical literacy. Moreover, the high-income group has a strong ability to bear transportation costs. The distance of transportation should not cause any hindrance to this group of people. Therefore, they use outdoor sports venues more frequently, which is more beneficial to the health of the high-income group. For low-income groups, the regression coefficient of public sports expenditure is not statistically significant, nor is it significant for middle-income groups. This indicates that the expansion of mass sports expenditure does not have a significant improvement effect on the health of residents in these two income groups. It indicates that the expenditure on mass sports during this period had the characteristic of being “pro-rich.”

**Table 4:** Results of the impact of public sports expenditure on the health of different income groups.

Variable	(1) High-income group	(2) Middle-income group	(3) Low-income group
Amateur	0.287 * (1.91)	0.278 (0.67)	0.785 (0.25)
Control variable	Yes	Yes	Yes
*N*	53,031	53,031	53,031
*R* ^2^	0.685	0.674	0.664

***, **, * represent significant at the 1, 5, and 10% levels, respectively.

#### 5.3.2 The impact of public sports expenditure on the health of groups with different educational levels

As shown in Table 5, public sports expenditure has also had different effects on the health of different educational groups. For residents without higher education, the regression coefficient of public sports expenditure is positive, and it has passed the 1% significance level test statistically. This indicates that the expansion of public sports expenditure can effectively improve the physical health of the group without higher education. For the group with higher education, although the regression coefficient of public sports expenditure is positive, it is not statistically significant, indicating that the expansion of mass sports expenditure will not have a significant impact on the health of residents with higher education. This is consistent with the research results of Lera-Lopez et al. (42). The reasons for this are, on the one hand, that people with higher education have a higher awareness of physical exercise and will voluntarily and proactively engage in it. Most of them will improve their health through private sector fitness centers, clubs and other forms of exercise, and have a relatively weak dependence on public sports. On the other hand, due to a deeper understanding of health, people tend to have a higher standard for evaluating their own health, which leads to an underestimation of health. Therefore, it shows that the health effect of public sports expenditure on the group with higher education is not significant.

**Table 5:** Results of the impact of public sports expenditure on the health of educated groups.

Variable	(1) A group with higher education	(2) The group without higher education
Amateur	0.377 (0.43)	0.391*** (7.82)
Control variable	Yes	Yes
N	53,031	53,031
R^2^	0.793	0.796

***, **, * represent significant at the 1, 5, and 10% levels, respectively.

#### 5.3.3 The impact of public sports expenditure on the health of residents of different age groups

To study the impact of public sports expenditure on different age groups, the samples were divided by age into youth (16–45 years old), middle age (46–60 years old), and the older adult (over 60 years old). As shown in Table 6, the impact of public sports expenditure on the physical health of residents of different ages varies. The health relationship between the youth group and the middle-aged group is not significant, while the public sports expenditure coefficient of the older adult group is positive and passes the significance level test. This is consistent with the viewpoints in the existing literature (43). For the older adult, those over 60 years old are basically in the retirement period. At this age, they have more leisure time and engage in entertainment, leisure and physical exercise more frequently, which can improve their health to a certain extent. Among the young population, a portion are students at school. At this stage, their health conditions are more influenced by school physical education. Moreover, due to their younger age and physiological characteristics, their health is on the rise. Another part is mainly engaged in various social work, under greater pressure from life and work, and has limited time to participate in sports activities. Therefore, the impact of sports expenditure on them may be relatively small. The middle-aged group has significant differences in social roles and living habits, which have a greater impact on their health. Considering these differences together cannot effectively demonstrate the health effects of public sports on them. This thus leads to the insignificance of the overall population and the impact of expenditure on their health.

**Table 6:** Results of the impact of public sports expenditure on the health of residents of different age groups.

Variable	(1) Youth group	(2) Middle-aged group	(3) Older adult group
Amateur	0.154 (0.24)	0.586 (0.68)	0.443*** (7.94)
Control variable	Yes	Yes	Yes
*N*	53,031	53,031	53,031
*R* ^2^	0.675	0.664	0.693

***, **, * represent significant at the 1, 5, and 10% levels, respectively.

#### 5.3.4 The impact of public sports expenditure on the health of urban and rural residents

As shown in Table 7, public sports expenditure has different effects on the physical health of rural and urban residents. The regression coefficient of public sports expenditure of rural residents is positive and statistically passes the significance level of 10%, indicating that public sports expenditure will have a relatively obvious improvement effect on the physical health of rural residents. However, the regression coefficient of public sports expenditure of urban residents is not statistically significant. The results of this study are also consistent with those of Zhao and Liu (2025), both of which demonstrate that the effect of sports expenditure is more significant in poverty-stricken areas (35). By comparing the expenditure on public sports between urban and rural areas, it is found that the expenditure on rural sports accounts for less than 20% of the total expenditure on mass sports, while the sports venues produced account for more than 50% of the total area. For a long time, there has been a scarcity of public sports resources in rural areas. Therefore, the newly added public expenditure on sports can quickly fill the gap and significantly improve the opportunities and conditions for residents to engage in sports activities, thereby generating high health benefits for residents and the impact can be easily captured. The sports infrastructure and public services in urban areas have been relatively well-developed, and the basic conditions for residents’ sports activities have been initially met. The newly added regular public expenditure may mainly be used for the maintenance of existing facilities or incremental upgrades, and its marginal improvement effect on the overall health level of residents is limited, manifested as statistically insignificant.

**Table 7:** Results of the impact of public sports expenditure on the health of urban and rural residents.

Variable	(1) Rural group	(2) Urban group
Amateur	0.483 * (1.83)	0.533 (0.81)
Control variable	Yes	Yes
*N*	53,031	53,031
*R* ^2^	0.562	0.598

***, **, * represent significant at the 1, 5, and 10% levels, respectively.

### 5.4 Robustness test

#### 5.4.1 Lag variable test

Due to the changes in the statistical classification of the data in the “China Sports Statistical Yearbook” since 2013, it cannot be used simultaneously with the data before 2013. And the CFPS data is tallied every 2 years. This article, after comparison and matching, can only conduct regression analysis on the data of 2014 and 2016, and there may be deviations in the time series. Therefore, in the model regression, a robustness test was conducted on the health of residents in 2016. Using the personal health data of residents in 2016, the explanatory variables were replaced with the public sports expenditure and the expenditure data of each category in 2015 and 2014 for regression with a lag of one period and a lag of two periods, in order to eliminate the differential effects brought by public sports expenditure in different years. Affirm the robustness of regression analysis. The results in Tables 8 and 9 show that the significance of the first and second phases of residents’ health lag has gradually weakened, and overall, it is considered to be relatively stable.

**Table 8:** Lagged one-period regression results.

Variable	(1)	(2)	(3)	(4)	(5)
Physical	0.146 (0.22)				
Amateur		0.716*** (5.78)			
Gym			0.164 (0.54)		
Athletic				0.115 (0.37)	
Else					0.011 (0.42)
Control variable	Yes	Yes	Yes	Yes	Yes
*N*	53,031	53,031	53,031	53,031	53,031
*R* ^2^	0.434	0.453	0.443	0.427	0.484

***, **, * represent significant at the 1, 5, and 10% levels, respectively.

**Table 9:** Results of lagged two-phase regression.

Variable	(1)	(2)	(3)	(4)	(5)
Physical	0.127 (0.23)				
Amateur		0.664*** (5.22)			
Gym			0.142 (0.31)		
Athletic				0.101 (0.31)	
Else					0.011 (0.27)
Control variable	Yes	Yes	Yes	Yes	Yes
*N*	53,031	53,031	53,031	53,031	53,031
*R* ^2^	0.427	0.422	0.431	0.422	0.432

***, **, * represent significant at the 1, 5, and 10% levels, respectively.

#### 5.4.2 Instrumental variable test

Considering the possibility of endogeneity issues due to omitted variables, this paper employs the instrumental variable method to alleviate the problem. Based on existing literature, the lagged one-period variable of the explanatory variable (*Physical*) is selected as the instrumental variable (*IV*) for public sports expenditure. This not only meets the requirement of correlation with the explanatory variable of public sports expenditure, but also satisfies the exogeneity requirement that it has no direct association with the dependent variable, the residents’ health level. The (1) and (2) columns of Table 10 present the test results of the instrumental variables. From the Wald F test result in column (1), it can be seen that the instrumental variables selected in this study do not have the problem of weak instruments. Combining the regression results in the second stage of column (2), it can be observed that after solving the endogeneity problem, the regression coefficient of public sports expenditure is 0.115, and it is significant at the 1% level, which is consistent with the previous results, indicating that the conclusion of this study is robust.

**Table 10:** Instrumental variable test.

Variable	(1)	(2)
IV	0.137*** (5.74)	
Physical		0.115*** (4.13)
Control variable	Yes	Yes
*N*	53,031	53,031
*R* ^2^	0.506	0.658
Wald *F*	118.32	

***, **, * represent significant at the 1, 5, and 10% levels, respectively.

## 6 Conclusions and suggestions

### 6.1 Conclusion

This study systematically analyzed the theoretical basis of public sports expenditure on residents’ health levels and conducted a micro-empirical analysis using the CFPS database to verify the research hypotheses. Research has found that public sports expenditure and mass sports expenditure can effectively enhance residents’ health, while the regression results of other types of expenditure are not satisfactory. Through the research on the impact of different groups, it is found that the high-income group is more significantly positively affected by public sports expenditure. Due to the insufficient awareness of sports among low-income groups and the fact that long working hours occupy their leisure time, the impact of public sports services on their health is not significant. By classifying the educational attainment, it is found that groups without higher education are more likely to benefit from the expenditure on public sports and thereby improve their own health. A regression study of the older adult, middle-aged and young age groups revealed that public sports expenditure significantly improved the health level of the older adult group. Through the analysis of urban and rural resident groups, it was found that the health improvement effect of rural residents’ expenditure on public sports is significant, while that of urban residents is relatively weak.

### 6.2 Suggestions

(1) Increase the proportion of mass sports expenditure in the total public sports expenditure. Empirical results show that competitive sports, sports venues, and other sports expenditures have not effectively promoted residents’ health. Therefore, the proportion of expenditure on competitive sports, sports venues and other sports should be appropriately reduced, while the proportion of expenditure on mass sports should be increased. In addition, it is suggested that the total amount of public sports expenditure be increased to promote the improvement of the public sports system and the construction of public sports infrastructure. This can effectively provide public sports services, improve the utilization rate of resources, and enhance the health effect of public sports expenditure on residents.

(2) Expand the opening of sports venues to residents and increase the utilization rate of sports venues by the public. Large sports venues have occupied the available land area in cities and reduced the accessibility for the public to engage in sports activities. The maintenance fees for large sports venues are relatively high, and they are generally rarely opened to the general public. This has led to a large number of sports venues being vacant, resulting in a waste of resources. However, the state’s subsidies for sports venues are limited, and the contradiction between supply and demand is difficult to resolve. Therefore, it is possible to attempt to introduce a market operation mechanism. Market mechanisms can make the operation of sports venues more flexible and broaden their development ideas. At the same time, under the background of market-oriented competition mechanism and public welfare, the threshold for public utilization can be lowered and the utilization rate of venues by the public can be improved.

(3) Develop public sports for the older adult to achieve healthy aging. The global population is gradually entering an aging stage at present. With the aggravation of aging, it is bound to increase the burden on the younger generation and also cause huge pressure on fiscal expenditure. For the older adult, their physical functions gradually decline, and they tend to rely more on medical treatment. Medical insurance expenditures will also increase, which will impose a financial burden. Therefore, more attention should be paid to the health issues of the older adult. Appropriately increase the number of sports activity venues for the older adult in communities. By regularly organizing small-scale sports activities for the older adult, we can enrich their lives and provide them with care in terms of both mental and physical aspects.

This paper studies the impact of the public service part of public sports expenditure on residents’ health. There are still certain limitations in the research process. For instance, (1) The scientific and technological, diplomatic expenditures and other aspects of the sports industry were not included in the research. Because these parts have relatively low expenditures and the pathways through which they affect residents are unclear. These expenditures can be included in future research. (2) There may also be other heterogeneous influences in the research. For instance, the sample of the higher education group can be further studied by subdividing it into different age groups and regions with different levels of economic development. This article was not included due to the difficulty in obtaining data. (3) This article may also have limitations in terms of causality. Therefore, in the future, with the update of data, further research can be conducted to explore whether public sports expenditure has further improved the health of residents.

## Data Availability

The data analyzed in this study is subject to the following licenses/restrictions: The dataset used in this study is available from the corresponding author upon request. Requests to access these datasets should be directed to https://www.isss.pku.edu.cn/cfps/.

## References

[1] Liu Z Zhang S Li L Hu B Liu R Zhao Z . Research on the construction and prediction of China's national fitness development index system under social reform. Front Public Health. (2022) 10:878515. doi: 10.3389/fpubh.2022.878515, 35651855 PMC9149160

[2] Shen Y Niu L Miao L. The impact of 15-minute fitness circles implemented in China on the public’s subjective well-being–an empirical analysis based on CGSS2021. Front Public Health. (2025) 13:1563722. doi: 10.3389/fpubh.2025.1563722, 40371288 PMC12077315

[3] Cheng M Zhang L Li D. Analysis of the coupled and coordinated development of sports and tourism industries and the driving factors. Sci Rep. (2023) 13:16725. doi: 10.1038/s41598-023-44025-6, 37794140 PMC10550941

[4] Li G Sun Q. Influence of fiscal expenditure of national fitness public services on the development of sports industry and national economy. J Shandong Sport Univ. (2025) 41:35–44. doi: 10.14104/j.cnki.1006-2076.2025.03.005

[5] General Administration of Sport of China. National sports venue statistical survey data in 2020. Available online at: https://www.sport.gov.cn/n315/n329/c20908193/content.html (2021).

[6] Tan TC. The transformation of China's National Fitness Policy: from a major sports country to a world sports power. Int J Hist Sport. (2015) 32:1071–84. doi: 10.1080/09523367.2015.1036240

[7] Zhou X Yan WY Li XT Li H Wu YZ Xu BC. Digital economy: an effective path for promoting residents' health in China. Front Public Health. (2023) 11:1303541. doi: 10.3389/fpubh.2023.1303541, 38074713 PMC10704149

[8] He YT Zhang YC Huang W Wang RN He LX Li B . Impact of digital economic development and environmental pollution on residents’ health: an empirical analysis based on 279 prefecture-level cities in China. BMC Public Health. (2023) 23:959. doi: 10.1186/s12889-023-15788-4, 37231366 PMC10212741

[9] Qin C Liu J Zhu Y Smith R Fang X. Bridging the urban–rural divide: the impact of medical insurance integration on health inequality in China. China Agric Econ Rev. (2025) 18:–125. doi: 10.1108/CAER-04-2024-0126

[10] Tang Y Fu R Noguchi H. Impact of medical insurance integration on reducing urban-rural health disparity: evidence from China. Soc Sci Med. (2024) 357:117163. doi: 10.1016/j.socscimed.2024.117163, 39121565

[11] Chen M Wang H. Does import trade liberalization worsen residents’ health? Evidence from China. Front Public Health. (2024) 12:1470338. doi: 10.3389/fpubh.2024.1470338, 39529706 PMC11550941

[12] Luo M Chen L. The impact of regional sports industry aggregation on residents’ health level in China. Sci Rep. (2024) 14:10928. doi: 10.1038/s41598-024-60564-y, 38740781 PMC11091125

[13] Ying M Wang S Bai C Li Y. Rural-urban differences in health outcomes, healthcare use, and expenditures among older adults under universal health insurance in China. PLoS One. (2020) 15:e0240194. doi: 10.1371/journal.pone.0240194, 33044992 PMC7549821

[14] Li J Yu S Xu Z. Does environmental pollution weaken the positive effect of government public expenditure on residents’ subjective well-being? A case study in China. Energy Environ. (2023) 34:927–45. doi: 10.1177/0958305X221079424

[15] Roessler M Schmitt J. Health system efficiency and democracy: a public choice perspective. PLoS One. (2021) 16:e0256737. doi: 10.1371/journal.pone.0256737, 34492045 PMC8423257

[16] Ruzima M Veerachamy P. The impact of public spending in education and health on human development in India. J Asia Pac Econ. (2023) 28:390–403. doi: 10.1080/13547860.2021.1952920

[17] Tong Y Wang H Zhu K Zhao H Qi Y Guan J . Satisfaction with community health education among residents in China: results from a structural equation model. Front Public Health. (2022) 10:905952. doi: 10.3389/fpubh.2022.905952, 35899165 PMC9309729

[18] Gu H Tan Q Guo Y He H Zhang Y. Family support, social security, commercial insurance, and aging anxiety among Chinese residents: a study based on the 2021 CGSS data. Front Public Health. (2025) 13:1577384. doi: 10.3389/fpubh.2025.1577384, 40265070 PMC12013337

[19] Liu D Chu Y. Impact of urban-rural resident basic medical insurance integration on individual social fairness perceptions: evidence from rural China. Front Public Health. (2024) 12:1408146. doi: 10.3389/fpubh.2024.1408146, 39267656 PMC11390400

[20] Wang D Li H Liu P. The impact of neighborhood environment and social interaction on the health of Chinese residents: empirical analysis from CGSS 2021. Front Public Health. (2025) 13:1547499. doi: 10.3389/fpubh.2025.1547499, 40401069 PMC12093411

[21] Pei Y Chen H Liu Z Hu H. Does environmental regulation improve residents' health? Evidence from China. Front Public Health. (2023) 11:973499. doi: 10.3389/fpubh.2023.973499, 36844837 PMC9945913

[22] Lu L Wei W. Influence of public sports services on residents’ mental health at communities level: new insights from China. Int J Environ Res Public Health. (2023) 20:1143. doi: 10.3390/ijerph20021143, 36673898 PMC9858637

[23] Wang D Zhang E Qiu P Hong X. Does increasing public expenditure on sports promote regional sustainable development: evidence from China. Front Public Health. (2022) 10:976188. doi: 10.3389/fpubh.2022.976188, 36211699 PMC9533120

[24] Wang Z Wei G. Effects of sports public goods on the health of the elderly: empirical evidence from China. Soc Work Public Health. (2022) 37:370–80. doi: 10.1080/19371918.2021.2017379 35007189

[25] Krekel C Kavetsos G Ziebarth NR. Passing on the flame: do mega sports events promote health behaviours? Soc Sci Med. (2025) 377:117921. doi: 10.1016/j.socscimed.2025.117921 40311500

[26] Steckenleiter C Lechner M Pawlowski T Schüttoff U. Do local expenditures on sports facilities affect sports participation? Econ Inq. (2023) 61:1103–28. doi: 10.1111/ecin.13161

[27] Liu S Zuo Y Chen S Law R Cui J. Short videos turn everyone into bearers of traditional sports and games: a mixed-methods study from China. Behav Sci. (2025) 15:637. doi: 10.3390/BS15050637, 40426415 PMC12109336

[28] Rich KA Nelson G Borgen-Flood T Pegoraro A. Regional policy and organizational fields in multi-level sport governance. European Sport Management Quarterly (2024). 24:51–71. doi: 10.1080/16184742.2023.2257715

[29] Brechot M Nüesch S Franck E. Does sports activity improve health? Representative evidence using local density of sports facilities as an instrument. Appl Econ. (2017) 49:4871–84. doi: 10.1080/00036846.2017.1296548

[30] Xue X Li Y. Will the construction of sports facilities nudge people to participate in physical exercises in China? The moderating role of mental health. Healthcare (Basel). (2023) 11:219. doi: 10.3390/healthcare11020219, 36673586 PMC9858653

[31] Kumar H Manoli AE Hodgkinson IR Downward P. Sport participation: from policy, through facilities, to users’ health, well-being, and social capital. Sport Manag Rev. (2018) 21:549–62. doi: 10.1016/j.smr.2018.01.002

[32] Wang DP Lin J. Does sports industry matter in human wellbeing: evidence from China? Front Public Health. (2022) 10:872506. doi: 10.3389/fpubh.2022.872506, 35400061 PMC8987153

[33] Zheng JY Luan LX Sun M. Does the national fitness policy promote national health?—an empirical study from China. Int J Environ Res Public Health. (2022) 19:9191. doi: 10.3390/ijerph19159191, 35954550 PMC9368402

[34] Wicker P Downward P Lera-López F. Does regional disadvantage affect health-related sport and physical activity level? A multi-level analysis of individual behaviour. Eur J Sport Sci. (2017) 17:1350–9. doi: 10.1080/17461391.2017.1376119, 28934600

[35] Zhao L Liu M. Does the improvement of financial inclusion enhance the distribution of sports resources between urban and rural areas? Fin Res Lett. (2025) 86:108407. doi: 10.1016/j.frl.2025.108407

[36] Ren C Wang C Zhang M. The antidepressant effect of physical exercise: evidence from China family panel studies. PLoS One. (2022) 17:e0274321. doi: 10.1371/journal.pone.0274321, 36201480 PMC9536592

[37] Sun H Cheng M Jiang F. Research on the influence mechanism of digital economy on the development of the sports and health industry in China. Sci Rep. (2025) 15:30595. doi: 10.1038/s41598-025-16641-x, 40835887 PMC12368095

[38] Cheng M Zhang L Zhang X Li Y. Does rural sports tourism promote the sustainable development of the destination?--based on quasi-experimental evidence of sports and leisure towns in China. J Clean Prod. (2025) 486:144537. doi: 10.1016/j.jclepro.2024.144537

[39] Liu Y Huo S. Measurement of health human capital and its economic effect in China. Humanit Soc Sci Commun. (2024) 11:1–10. doi: 10.1057/s41599-024-03060-y

[40] Wang H Dai X Wu J Wu X Nie X. Influence of urban green open space on residents’ physical activity in China. BMC Public Health. (2019) 19:1093. doi: 10.1186/s12889-019-7416-7, 31409316 PMC6693084

[41] Chekroud SR Gueorguieva R Zheutlin AB Paulus M Krumholz HM Krystal JH . Association between physical exercise and mental health in 1· 2 million individuals in the USA between 2011 and 2015: a cross-sectional study. Lancet Psychiatry. (2018) 5:739–46. doi: 10.1016/S2215-0366(18)30227-X, 30099000

[42] Lera-Lopez F Wicker P Downward P. Does government spending help to promote healthy behavior in the population? Evidence from 27 European countries. J Public Health. (2016) 38:e5–e12. doi: 10.1093/pubmed/fdv071, 26054911

[43] Li Y Song H. The association between sports social capital and cognitive health: a longitudinal study of middle-aged and elderly adults in China. SSM Popul Health. (2025) 30:101778. doi: 10.1016/j.ssmph.2025.101778, 40212736 PMC11982950

